# HASE: Framework for efficient high-dimensional association analyses

**DOI:** 10.1038/srep36076

**Published:** 2016-10-26

**Authors:** G. V. Roshchupkin, H. H. H. Adams, M. W. Vernooij, A. Hofman, C. M. Van Duijn, M. A. Ikram, W. J. Niessen

**Affiliations:** 1 Department of Radiology and Nuclear Medicine, Erasmus MC, Rotterdam, Netherlands; 2 Department of Medical Informatics, Erasmus MC, Rotterdam, Netherlands; 3 Department of Epidemiology, Erasmus MC, Netherlands; 4 Department of Neurology, Erasmus MC, Rotterdam, Netherlands; 5 Faculty of Applied Sciences, Delft University of Technology, Delft, Netherlands

## Abstract

High-throughput technology can now provide rich information on a person’s biological makeup and environmental surroundings. Important discoveries have been made by relating these data to various health outcomes in fields such as genomics, proteomics, and medical imaging. However, cross-investigations between several high-throughput technologies remain impractical due to demanding computational requirements (hundreds of years of computing resources) and unsuitability for collaborative settings (terabytes of data to share). Here we introduce the HASE framework that overcomes both of these issues. Our approach dramatically reduces computational time from years to only hours and also requires several gigabytes to be exchanged between collaborators. We implemented a novel meta-analytical method that yields identical power as pooled analyses without the need of sharing individual participant data. The efficiency of the framework is illustrated by associating 9 million genetic variants with 1.5 million brain imaging voxels in three cohorts (total N = 4,034) followed by meta-analysis, on a standard computational infrastructure. These experiments indicate that HASE facilitates high-dimensional association studies enabling large multicenter association studies for future discoveries.

Technological innovations have enabled the large-scale acquisition of biological information from human subjects. The emergence of these big datasets has resulted in various ‘omics’ fields. Systematic and large-scale investigations of DNA sequence variations (genomics)^1^, gene expression (transcriptomics)^2^, proteins (proteomics)^3^, small molecule metabolites (metabolomics)^4^, and medical images (radiomics)^5^, among other data, lie at the basis of many recent biological insights. These analyses are typically unidimensional, i.e. studying only a single disease or trait of interest.

Although this approach has proven its scientific merit through many discoveries, jointly investigating multiple big datasets would allow for their full exploitation, as is increasingly recognized throughout the ‘omics’ world^5^,^6^,^7^,^8^. However, the high-dimensional nature of these analyses makes them challenging and often unfeasible in current research settings. Specifically, the computational requirements for analyzing high-dimensional data are far beyond the infrastructural capabilities for single sites. Furthermore, it is incompatible with the typical collaborative approach of distributed multi-site analyses followed by meta-analysis, since the amount of generated data at every site is too large to transfer.

Some studies have attempted to combine multiple big datasets^5^,^8^,^9^,^10^, but these methods generally rely on reducing the dimensionality or making assumptions to approximate the results, which leads to a loss of information.

Here we present the framework for efficient high-dimensional association analyses (HASE), which is capable of analyzing high-dimensional data at full resolution, yielding exact association statistics (i.e. no approximations), and requiring only standard computational facilities. Additionally, the major computational burden in collaborative efforts is shifted from the individual sites to the meta-analytical level while at the same time reducing the amount of data needed to be exchanged and preserving participant privacy. HASE thus removes the current computational and logistic barriers for single- and multi-center analyses of big data.

## Results

### Overview of the methods

The methods are described in detail in the Methods. Essentially, HASE implements a high-throughput multiple linear regression algorithm that is computationally efficient when analyzing high-dimensional data of any quantitative trait. Prior to analysis, data are converted to an optimized storage format to reduce reading and writing time. Redundant calculations are removed and the high-dimensional operations are simplified into a set of matrix operations that are computationally inexpensive, thereby reducing overall computational overhead. While deriving summary statistics (e.g., beta coefficients, p-values) for every combination in the high-dimensional analysis would be computationally feasible at individual sites with our fast regression implementation, it would be too large to share the intermediate results (>200 GB per thousand phenotypes) in a multi-center setting. Therefore, extending from a recently proposed method, partial derivatives meta-analysis^11^, we additionally developed a method that generates two relatively small datasets (e.g. 5 GB for genetics data of 9 million variants and 20 MB of thousand phenotypes for 4000 individuals) that are easily transferred and can subsequently be combined to calculate the full set of summary statistics, without making any approximation. This meta-analysis method additionally reduces computational overhead at individual sites by shifting the most expensive calculation to the central site. The total computational burden thus becomes even more efficient relative to conventional methods with additional sites. The HASE software is freely available from our website www.imagene.nl/HASE/.

### Comparison of complexity and speed

We compared the complexity and speed of HASE with a classical workflow, based on linear regression analyses with PLINK (version 1.9)^12^ followed by meta-analysis with METAL^13^; two of the most popular software packages for these tasks.

Table 1 shows that HASE dramatically reduces the complexity for the single site analysis and data transfer stages. For conventional methods, the single site analysis and data transfer have a multiplicative complexity (dependent on the number of phenotypes and determinants), whereas this is only additive for HASE. Our approach requires 3.500-fold less data to transfer for a high-dimensional association study. Additionally, the time for single site analysis does not increase significantly from analyzing a single phenotype to a million phenotypes (Table 1). This is due to the fact that speed is determined by the highest number of either the determinants or phenotypes. Therefore, in this case with nine million genetic variants, the complexity of ***O***(***n***_***i***_***n***_***p***_) is the primary factor influencing the speed, whereas ***O***(***n***_***i***_***n***_***t***_) plays a secondary role.

This drastic increase in performance is made possible through the shift of the computationally most expensive regression operation to the meta-analytical stage. For the meta-analytical stage, the HASE complexity is therefore slightly higher. However, it outperforms the classical meta-analysis using METAL (total computation time reduced 35 times), owing to the efficient implementation of our algorithm.

**Figure 1:**
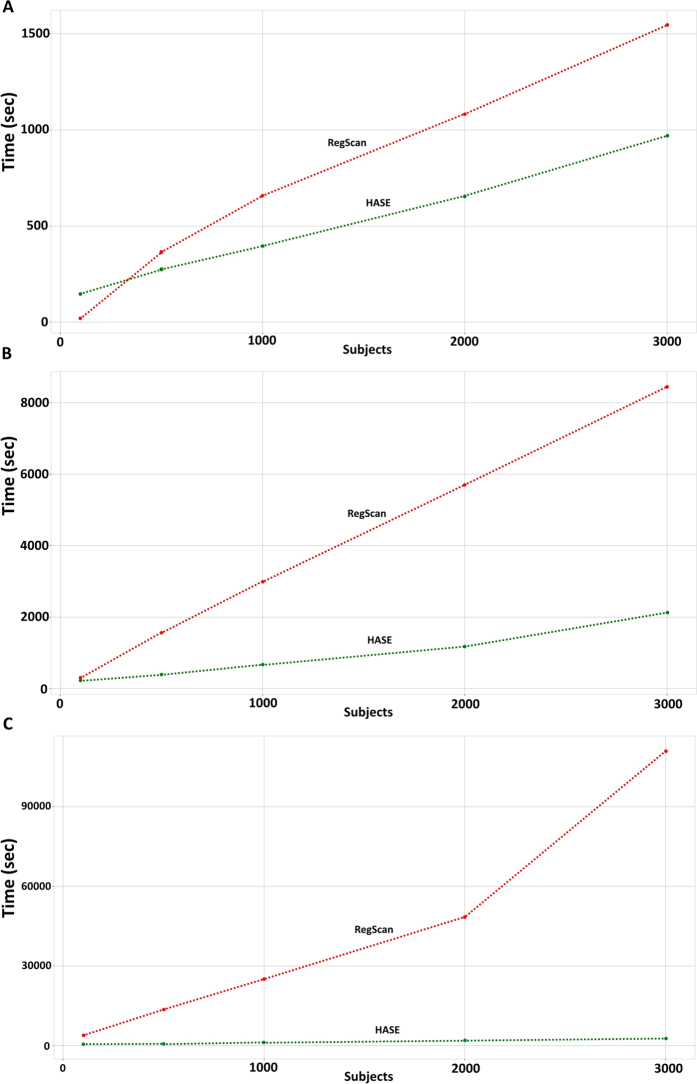
Analysis time (HASE versus RegScan) with 2.172.718 variants. (**A**)– for 1 phenotype; (**B**)– for 100 phenotypes; **(C)-** for 1000 phenotypes.

Additionally, HASE can be used as a standard tool for high-dimensional association studies of a single site, i.e without subsequent meta-analysis or to prepare summary statistics for sharing with the central site as in a classical workflow. Although PLINK is a very popular tool for association analysis, it is not optimized for high-dimensional data sets. Therefore we compared the speed of such analyses to the recently developed tool RegScan^14^, which was developed for doing GWAS on multiple phenotypes and outperformed state-of-the-art methods. We conducted several experiments within the Rotterdam Study by varying the number of phenotypes and subjects, while keeping the number of variants fixed at 2.172.718 since the complexity of both programs is linear with respect to number of variants. HASE outperformed RegScan and the difference becomes larger for increasing numbers of subjects and phenotypes (Fig. 1).

### Application to real data

We used HASE to perform a high-dimensional association study in 4,034 individuals from the population-based Rotterdam Study. In this proof of principle study, we relate 8,723,231 million imputed genetic variants to 1,534,602 million brain magnetic resonance imaging (MRI) voxel densities (see Supplementary Note). The analysis was performed on a small cluster of 100 CPUs and took 17 hours to complete.

**Figure 2:**
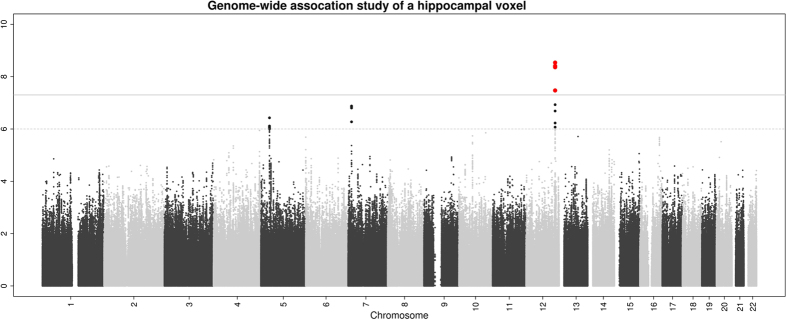
Manhattan plot of the hippocampus voxel with the most significant association after screening all 7030 hippocampal voxels. The most significant association (rs77956314; p = 3 × 10^−9^) corresponded to a previously identified locus on chromosome 12q24. Such voxel-wise hippocampus screening would take less than 8 hours on standard laptop.

To demonstrate the potential of such high-dimensional analyses, we screened all genetic association results for both hippocampi (7,030 voxels) and identified the voxel with the lowest p-value. The most significant association (rs77956314; p = 3 × 10^−9^) corresponded to a locus on chromosome 12q24 (Fig. 2), which was recently discovered in a genome-wide association study of hippocampal volume encompassing 30,717 participants^15^.

**Figure 3:**
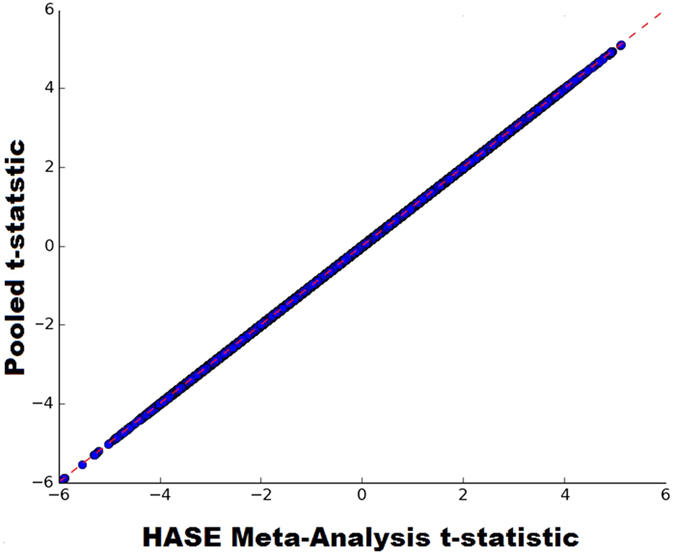
Correlation plot of voxel GWAS t-statistic estimated from pooled together data and voxel GWAS t-statistic estimated from meta-analysis of partial derivatives and encoded matrix. It took 40 min for single site to pre-compute data instead of 280 years to compute summary statistics.

Additionally, we performed the high-dimensional association studies separately in three subcohorts of the Rotterdam Study (RSI = 841, RSII = 1003, RSIII = 2190, Supplementary Notes) and meta-analyzed the results using the HASE data sharing approach, as a simulation of a standard multicenter association study. This experiment required two steps. First, for each subcohort we generated intermediate data (matrices **A**, **B** and **C** from the Methods section). It took on average 40 minutes on a single CPU for all genetic variants and voxels. Second, the meta-analysis, which consist of merging intermediate data and running regressions, was performed on the same cluster and took 17 hours to complete using 100 cores. We compared the association results of the pooled analysis with the meta-analysis. Figure 3 shows that the results are identical as it was predicted by theory (see Methods). We would like to point out that for the classical approach with inverse-variance meta-analysis such an experiment would be not possible to conduct, as it would require generating and sharing hundreds of terabytes of summary statistics.

## Discussion

We describe a framework that allows for (i) computationally-efficient high-dimensional association studies within individual sites using standard computational infrastructure and (ii) facilitates the exchange of compact summary statistics for subsequent meta-analysis for association studies in a collaborative setting. Using HASE, we performed a genome-wide and brain-wide search for genetic influences on voxel densities (more than 1.5 million GWAS analysis in total), and illustrate both its feasibility and potential for driving scientific discoveries.

A large improvement in efficiency comes from the reduced computational complexity. High-dimensional analyses contain many redundant calculations, which were removed in the HASE. Also, we were able to further increase efficiency by simplifying the calculations to a set of matrix operations, which are computationally inexpensive, compared to conventional linear regression algorithms. Furthermore, the implementation of partial derivatives meta-analysis allowed us to greatly reduce the size of the summary statistics that need to be shared for performing a meta-analysis. Another advantage of this approach is that it only needs to calculate the partial derivatives for each site instead of the parameter estimates (i.e., beta coefficients and standard errors). This enabled us to develop within HASE a reduction approach that encodes data prior to exchange between sites, while yielding the exact same results after meta-analysis as if the original data were used. The encoding is performed such that tracing back to original data is impossible. This guarantees protection of participant privacy and circumvents restrictions on data sharing that are unfortunately common in many research institutions.

When using HASE, it is first necessary to convert the multi-dimensional data to ≪HDF5^16^≫ format that is optimized for fast reading and writing. This particular format is not dependent on the architecture of the file system and can therefore be implemented on a wide range of hardware and software infrastructures. To facilitate this initial conversion step, we have built-in tools within the HASE framework for processing common file format of such big data. HDF5 allows direct access to the data matrix row/column from the disk through an index without reading the whole file(s) into memory. Additionally, it requires much less disk space to store data (Supplementary Notes). This is easily generalizable to other large omics datasets in general and we foresee this initial conversion step not to form an obstacle for researchers to implement HASE.

Alternative methods for solving the issues with high-dimensional data take one of two approaches. One approach is to reduce the dimensionality of the big datasets by summarizing the large amount of data into fewer variables^2^. Although this increases the speed, it comes at the price of losing valuable information, which these big data were primarily intended to capture. The second approach is to not perform a full analysis of all combinations of the big datasets, but instead make certain assumptions (e.g., a certain underlying pattern, or a lack of dependency on potential confounders) that allow for using statistical models that require less computing time. Again, this is a tradeoff between speed and accuracy, which is not necessary in the HASE framework, where computational efficiency is increased without introducing any approximations.

Unidimensional analyses of big data, such as genome-wide association studies, have already elucidated to some extent the genetic architecture of complex diseases and other traits of interest^1^,^17^,^18^,^19^, but much remains unknown. Cross-investigations between multiple big datasets potentially hold the key to fulfill the promise of big data in understanding of biology^7^. Using the HASE framework to perform high-dimensional association studies, this hypothesis is now testable.

## Methods

### HASE

In high-dimensional associations analyses we test the following simple regression model:


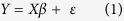


where **Y** is a **n**_**i**_ × **n**_**p**_ matrix of phenotypes of interest, **n**_**i**_denotes the number of samples in the study, **n**_**p**_ the number of phenotypes of interest, and ε denotes the residual effect. **X** is a three dimensional matrix **n**_**i**_ × **n**_**c**_ × **n**_**t**_ of independent variables, with **n**_**c**_ representing the number of covariates, such as the intercept, age, sex and, for example genotype as number of alleles, and **n**_**t**_the number of independent determinants.

In association analyses we are interested in estimating the p-value to test the null hypothesis that β = 0. The p-values can be directly derived from the t-statistic of our test determinants. We will rewrite the classical equation for calculating t-statistics for our multi-dimensional matrices, which will lead to a simple matrix form solution for high-dimensional association analysis:


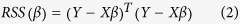



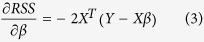



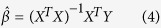



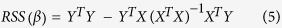



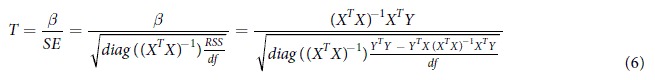


where **T** is **n**_**p**_ × **n**_**c**_ × **n**_**t**_ matrix of t-statistics and **df** is degree of freedom of our regression model. Let’s define 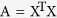, 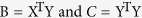, so that we can write our final equation for t-statistics:


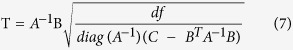


The result of this derivation is that, rather than computing all combinations of covariates and independent determinants, we only need to know three matrices: A, B and C, to calculate t-statistics and perform the full analysis. These results will be used in the section about meta-analysis.

The most computationally expensive operations here are the two multi-dimensional matrix multiplications (***A***^−1^**B**) and (**B**^***T***^***A***^−1^**B**), where ***A***^−1^ is a three dimensional matrix **n**_**c**_ × **n**_**c**_ × **n**_**t**_ and 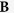
is three dimensional matrix **n**_**c**_ × **n**_**p**_ × **n**_**t**_. Without knowledge of the data structure of these matrices, the simplest way to write the results of their multiplication would be to use Einstein’s notation for tensor multiplication:


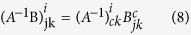



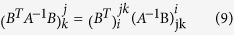


*where*


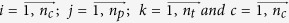


As you can see, the result is two matrices of **n**_**c**_ × **n**_**p**_ × **n**_**t**_ and **n**_**p**_ × **n**_**t**_ elements respectively. Despite the seemingly complex notation, the first matrix just represents the beta coefficients for all combinations of covariates (**n**_**c**_ by **n**_**p**_ × **n**_**t**_ combinations) and the second is fitting values of the dependent variable for every test (**n**_**p**_ × **n**_**t**_ independent determinants).

However, insight into the data structure of **A** and **B** can dramatically reduce the computational burden and simplify operations. First of all, matrix **A** depends only on the covariates and number of determinants, making it unnecessary to compute it for every phenotype of interest, so we just need to calculate it once. Additionally, only the last covariate (i.e., the variable of interest) is different between tests, meaning that the **(n**_**p**_**−1)** × **(n**_**p**_**−1)** × **n**_**t**_ part of matrix **A** remains constant during high-dimensional analyses. Matrix **B** consists of the dot product of every combination of the covariate and phenotype of interest. However, as we mentioned before, there are only (**n**_**t**_ **+ n**_**c**_ **1)** different covariates, and thus we can split matrix **B** in two low dimensional matrices: the first includes dot products of non-tested covariates - (**n**_**c**_**−1)** × **n**_**p**_ elements; the second includes the dot products only of the tested covariates - **n**_**p**_ × **n**_**t**_ elements. Removing all these redundant calculations reduces the complexity of this step from **O(n**_**c**_^**2**^ **· n**_**i**_ **·** **n**_**p**_ **·** **n**_**t**_**) to O(n**_**p**_ **· n**_**t**_). All this allows us to achieve a large gain in computational efficiency and memory usage. In Fig. 3 we show a 2D schematic representation of these two matrices for standard genome association study with the covariates being an intercept, age, sex, and genotype. This example could be easily extrapolated to any linear regression model.

Applying the same splitting operation to ***B***^***T***^ it is possible to simplify tensor multiplication equation (8, 9) to a low-dimensional matrix operation and rewrite the equation for t-statistics:


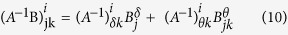



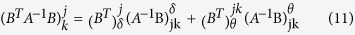



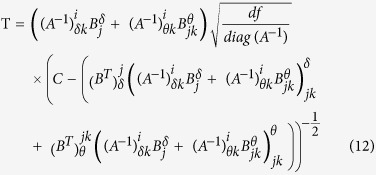


Then, to compute t-statistics for high-dimensional association analyses we just need to perform several matrix multiplications.

### Meta-analysis

In classical meta-analysis, summary statistics such as beta coefficients and p-values are exchanged between sites. For 1.5 million phenotypes, this would yield around 400TB of data at each site, making data transfer to a centralized site impractical.

**Figure 4:**
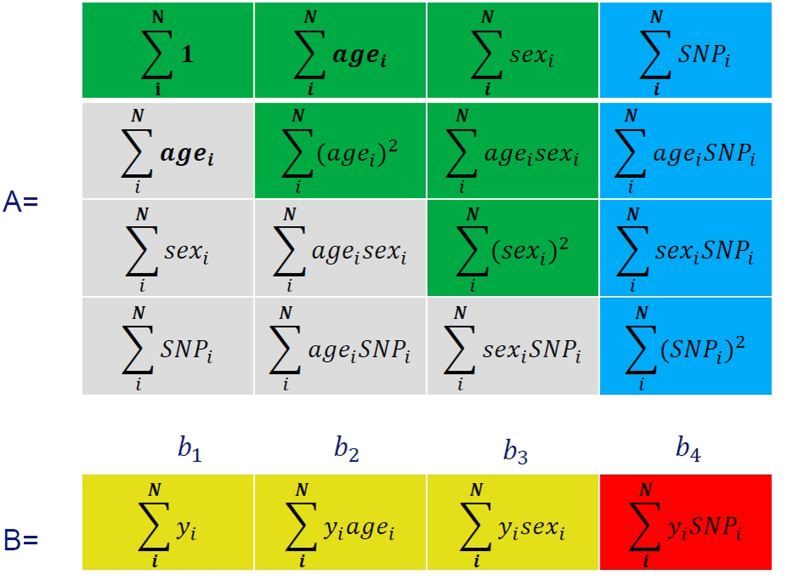
Explanation of the achieved speed reduction in HASE framework by removing redundant computations. In HASE multi-dimensional (**A**,**B**) matrices need to be calculated to perform GWAS studies. In the figure grey color means elements are parts of the matrix that are not necessary to calculate, as the **A** matrix is symmetric. The green color indicates elements that need to be calculated only once. Blue elements only have to be calculated for every SNP and yellow only for every phenotype. The red color indicates the most computationally expensive element, which needs to be calculated for every combination of phenotype and genotype. N denotes the number subjects in study.

In the previous section we showed that, to compute all statistics for an association study, we just need to know the **A, B** and **C** matrices. As we demonstrated before^11^, by exchanging these matrices between sites, it is possible to gain the same statistical power as with a pooled analysis, without sharing individual participant data, because these matrices consist of aggregate data (Fig. 4). However, in high-dimensional association analyses, matrix **B** grows very fast, particularly the part that depends on the number of determinants and phenotypes (**b**_**4**_ in Fig. 3).

If **Y** is a **n**_**i**_ × **n**_**p**_ matrix of phenotypes of interest and **G** is a **n**_**i**_ × **n**_**t**_ matrix of determinants which we want to test (e.g., a genotype matrix in GWAS), then **b**_**4**_ = **Y**^**T**^ × **G.** These two matrices, **Y** and **G**, separately are not so large, but their product matrix has **n**_**p**_ × **n**_**t**_ elements, which in a real application could be 10^6^ × 10^7^  = 10^13^ elements and thus too large to share between sites. We propose to create a random **n**_**i**_ × **n**_**i**_ nonsingular square matrix **F** and calculate its inverse matrix **F**^**−1**^. Then by definition **F** × **F**^**−1**^ = **I**, where **I** is a **n**_**i**_ × **n**_**i**_ elements identity matrix with ones on main diagonal and zeros elsewhere. Using this property, we can rewrite the equation for **b**_**4**_:


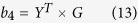



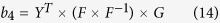



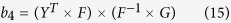



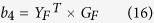


*where Y*_*F*_ *and G*_*F*_ *are matrices carrying phenotypic and deeterminant information in encoded form respectively*

Therefore, instead of transferring TBs of intermediate statistics (**b**_**4**_), each side just needs to compute **A, C, Y**_**F**_ **and G**_**F**_.

Sharing just the encoded matrices does not provide information on individual participants and without knowing matrix **F** it is impossible to reconstruct the real data. However, it will be possible to calculate **b**_**4**_, perform a high-dimensional meta-analysis, and avoid problems with data transfer. Additionally, this method dramatically reduces computation time by shifting all complex computations to central site, where the HASE regression algorithm should be used to handle the association analysis in time efficient way.

### Availability

Framework for efficient high-dimensional association analyses (HASE), https://github.com/roshchupkin/HASE/; description of the framework and protocol for meta-analysis, www.imagene.nl/HASE.

## Additional Information

**How to cite this article**: Roshchupkin, G. V. *et al*. HASE: Framework for efficient high-dimensional association analyses. *Sci. Rep.* **6**, 36076; doi: 10.1038/srep36076 (2016).

**Publisher’s note:** Springer Nature remains neutral with regard to jurisdictional claims in published maps and institutional affiliations.

## Supplementary Material

Supplementary Information

## Figures and Tables

**Table 1:** Comparison of complexity and speed between the HASE framework and a classical workflow.

Stage	Complexity^c^		Time^a^^,^^b^ (hours)			
	Classical workflow	HASE	*n*_*p*_ = 1		*n*_*p*_ = 10^6^	
			Classical workflow	HASE	Classical workflow	HASE
**Single site analysis**	**O(*****n***_***i***_***n***_***p***_***n***_***t***_)	**max (*****O***(***n***_***i***_***n***_***p***_), ***O***(***n***_***i***_***n***_***t***_))	2.46	0.63	2.46 × 10^6^	0.70
**Data transfer**	**O(*****n***_***p***_***n***_***t***_)	***O***(***n***_***i***_***n***_***p***_** + *****n***_***i***_***n***_***t***_)	0.04	0.07*	4 × 10^4^	11.6
**Meta-Analysis**	**O(*****n***_***p***_***n***_***t***_)	***O***(***n***_***i***_***n***_***p***_***n***_***t***_)	0.06	0.03	6 × 10^4^	1.7 × 10^3^

^a^Based on a model with three covariates and 9 million genetic variants, for a total of 4034 participants from three sites. For the classical workflow we used the PLINK software for single site analysis and METAL for the meta-analysis.

^b^For single site analysis and meta-analysis the time is given in CPU hours; for the data transfer stage this is in hours using an average network speed of 10 Mbps.

^c^Complexity for CPU hours is given in terms of classical computation time complexity; complexity for data transfer is shown in terms of how the size of the to be transferred data depends on the size of the input data.

^*^This time is derived from the transfer of partial derivatives only, because for an association analysis with relatively few phenotypes it is not necessary to transfer encoded data.

***n***_*i*_ - number of individuals in the study; ***n***_*p*_ - number of phenotypes of interest; ***n***_*t*_ - number of tests (genetic variants); ***N***_**s**_ - number of sites in the meta-analysis. In standard analysis ***n***_*i*_ ≪ ***n***_***p***_ and ***n***_*i*_ ≪ ***n***_*t*_.

## References

[b1] Wood A. R. . Defining the role of common variation in the genomic and biological architecture of adult human height. Nat. Genet. 46, 1173–1186 (2014). 25282103 10.1038/ng.3097 PMC4250049

[b2] Goel P., Kuceyeski A., Locastro E. & Raj A. Spatial patterns of genome-wide expression profiles reflect anatomic and fiber connectivity architecture of healthy human brain. Hum. Brain Mapp. 35, 4204–4218 (2014). 24677576 10.1002/hbm.22471 PMC4283562

[b3] Stunnenberg H. G. & Hubner N. C. Genomics meets proteomics: Identifying the culprits in disease. Hum. Genet. 133, 689–700 (2014). 24135908 10.1007/s00439-013-1376-2 PMC4021166

[b4] Krumsiek J. . Mining the Unknown: A Systems Approach to Metabolite Identification Combining Genetic and Metabolic Information. PLoS Genet. 8, e1003005 (2012). 23093944 10.1371/journal.pgen.1003005 PMC3475673

[b5] Parmar C. . Radiomic feature clusters and Prognostic Signatures specific for Lung and Head &amp; Neck cancer. Sci. Rep. 5, 11044 (2015). 26251068 10.1038/srep11044 PMC4937496

[b6] Medland S. E., Jahanshad N., Neale B. M. & Thompson P. M. Whole-genome analyses of whole-brain data: working within an expanded search space. Nat. Publ. Gr. 17, 791–800 (2014). 10.1038/nn.3718 PMC4300949 24866045

[b7] Robinson M. R., Wray N. R. & Visscher P. M. Explaining additional genetic variation in complex traits. Trends Genet. 30, 124–132 (2014). 24629526 10.1016/j.tig.2014.02.003 PMC4639398

[b8] Zou F. . Brain Expression Genome-Wide Association Study (eGWAS) Identifies Human Disease-Associated Variants. PLoS Genet. 8, e1002707 (2012). 22685416 10.1371/journal.pgen.1002707 PMC3369937

[b9] Stein J. L. . Voxelwise genome-wide association study (vGWAS). Neuroimage 53, 1160–1174 (2010). 20171287 10.1016/j.neuroimage.2010.02.032 PMC2900429

[b10] Huang M. . FVGWAS: Fast voxelwise genome wide association analysis of large-scale imaging genetic data. Neuroimage 118, 613–627 (2015). 26025292 10.1016/j.neuroimage.2015.05.043 PMC4554832

[b11] Adams H. H. H. . Partial derivatives meta-analysis: pooled analyses without sharing individual participant data. BioRxiv (2016).

[b12] Purcell S. . PLINK: a tool set for whole-genome association and population-based linkage analyses. Am. J. Hum. Genet. 81, (2007). 10.1086/519795 PMC1950838 17701901

[b13] Willer C. J., Li Y. & Abecasis G. R. METAL: Fast and efficient meta-analysis of genomewide association scans. Bioinformatics 26, 2190–2191 (2010). 20616382 10.1093/bioinformatics/btq340 PMC2922887

[b14] Haller T. RegScan: A GWAS tool for quick estimation of allele effects on continuous traits and their combinations. Brief. Bioinform. 16, 39–44 (2013). 24008273 10.1093/bib/bbt066 PMC4293375

[b15] Hibar D. P. . Common genetic variants influence human subcortical brain structures. Nature 8 (2015). 10.1038/nature14101 PMC4393366 25607358

[b16] The HDF5 Group, “HDF5 File Format Specification Version 3.0” (2006) https://support.hdfgroup.org/HDF5/doc/H5.format.html.

[b17] Locke A. E. . Genetic studies of body mass index yield new insights for obesity biology. Nature 518, 197–206 (2015). 25673413 10.1038/nature14177 PMC4382211

[b18] Purcell S. M. . Common polygenic variation contributes to risk of schizophrenia and bipolar disorder. Nature 460, 748–752 (2009). 19571811 10.1038/nature08185 PMC3912837

[b19] Polychronakos C. & Alriyami M. Diabetes in the post-GWAS era. Nat. Genet. 47, 1373–1374 (2015). 26620109 10.1038/ng.3453

