# Supplementary material for: HASE: Framework for efficient high-dimensional association analyses
Source: Sci Rep. 2016 Oct 26;6:36076. doi: 10.1038/srep36076 (PMC5080584; doi:10.1038/srep36076)
Supplement: Supplementary Information [file srep36076-s1.pdf]

**SUPPLEMENTARY NOTE**

# HASE: Framework for efficient high-dimensional association analyses


**AUTHORS**

G.V. Roshchupkin[2,4] , H.H.H. Adams[1,2] , M.W. Vernooij[1,2], A. Hofman[1], C.M. Van Duijn[1], M.A. Ikram[1,2,5*] and W.J. Niessen[2,3,4*]

**AFFILIATIONS**

[1] Department of Epidemiology, Erasmus MC, Netherlands,

[2] Department of Radiology, Erasmus MC, Rotterdam, Netherlands

[3] Faculty of Applied Sciences, Delft University of Technology, Delft, Netherlands

[4] Department of Medical Informatics, Erasmus MC, Rotterdam, Netherlands

[5] Department of Neurology, Erasmus MC, Rotterdam, Netherlands

* starred authors jointly supervised this work


**Word count manuscript: 694**

**References: 6**

**Figures: 1**


**Correspondence:**

M.A. Ikram, MD, PhD, Department of Epidemiology, Erasmus MC

**Phone nr.:** +31 10 70 43930 / **Fax nr.:** +31 10 70 43489 / **E-mail:**m.a.ikram@erasmusmc.nl

W. J. Niessen, PhD, Department of Medical Informatics and Radiology, Erasmus MC
**Phone nr.:** +31 10 70 43050 / **Fax nr.:** +31 10 70 44722 / **E-mail:** w.niessen@erasmusmc.nl
Rotterdam, Dr. Molewaterplein 50, 3015 GE, Rotterdam, the Netherlands.

### *Study Population*

The Rotterdam Study is an ongoing population-based cohort study in the Netherlands investigating diseases in the elderly and currently consists of 14,926 residents of Rotterdam who were aged 45 years or more at baseline [1,2]. The initial cohort was started in 1990 and expanded in 2000 and 2005. The whole population is subject to a set of multidisciplinary examinations every four years. MRI was implemented in 2005 and 5430 persons scanned until 2011 were eligible for this study. We excluded individuals with incomplete acquisitions, scans with artifacts hampering automated processing, participants with MRI-defined cortical infarcts, and subjects with dementia or stroke at the time of scanning. This resulted in a final study population of 4071 non-demented persons with information available on both genome-wide genotyping and MRI data. The Medical Ethics Committee of the Erasmus MC, University Medical Center Rotterdam and the review board of the Netherlands Ministry of Health, Welfare and Sports both approved the study. Informed consent was obtained from all subjects.

### *Imputation of genotypes*

The Illumina 550K and 550K duo arrays were used for genotyping. Samples with low call rate (<97.5%), with excess autosomal heterozygosity (>0.336) or with sex-mismatch were excluded, as were outliers identified by the identity-by-state clustering analysis (outliers were defined as being >3 standard deviation (SD) from population mean or having identity-by-state probabilities >97%). A set of genotyped input SNPs with call rate >98%, MAF >0.001 and Hardy–Weinberg equilibrium (HWE) P-value > $10^{-6}$ was used for imputation. The Markov Chain Haplotyping (MACH) package version 1.0 software (Imputed to plus strand of NCBI build 37, 1000 Genomes phase I version 3) and minimac version 2012.8.6 were used for imputation.

### *MRI data*

From August 2005 onwards, a dedicated 1.5 Tesla MRI scanner (GE Healthcare, Milwaukee, Wisconsin, USA) is operational in the Rotterdam Study research center in Ommoord. This scanner is operated by trained research

technicians and all imaging data are collected according to standardized imaging protocols[2]. Brain MRI scans included a high-resolution 3D T1-weighted fast RF spoiled gradient recalled acquisition in steady state with an inversion recovery pre-pulse (FASTSPGR-IR) sequence with thin slices (voxel size<1mm$^3$)[2]*Image processing*

Voxel based morphometry (VBM) was performed according to an optimized VBM protocol [3]. First, all T1-weighted images were segmented into supratentorial gray matter (GM), white matter (WM) and cerebrospinal fluid (CSF) using a previously described k-nearest neighbor (kNN) algorithm, which was trained on six manually labeled atlases [4]. FSL software [5] was used for VBM data processing. First, all GM density maps were non-linearly registered to the standard GM probability template. For this study we chose the ICBM MNI152 GM template (Montreal Neurological Institute) with a 1x1x1 mm$^3$ voxel resolution. The MNI152 standard-space T1-weighted average structural template is derived from 152 structural images, which have been warped and averaged into the common MNI152 co-ordinate system after high-dimensional nonlinear registration.

A spatial modulation procedure was used to avoid differences in absolute GM volume due to the registration. This involved multiplying voxel density values by the Jacobian determinants estimated during spatial normalization. To gain more statistic power and decrease signal to noise ratio, all images were smoothed using a 3mm (FWHM 8mm) isotropic Gaussian kernel.

***Statistical analysis***

Linear regression models were fitted with voxel values of GM modulation density as the dependent variable and age, sex, and the number of minor alleles as independent variables. In total 1,534,602 voxels were processed.

*Comparison of data formats*

We compared HDF5[6] HASE format to .bed and .bgen. for the same genetic data (the subsets of people from Rotterdam Study and 2.172.718 variants) which we used in experiments from section "**Comparison of complexity and speed"** of the manuscript.

As can be seen in **Figure 1** HDF5 format requires much less disk space to store data and with increasing number of subjects the difference becomes more significant (2.8Gb versus 498Mb for 3000 subjects). Additionally, the HASE framework allows for performing genome-wide association studies, but is certainly not limited to genetic data. While formats such .bed and .bgen are optimized to store allele info, HDF5 does not use any a prior knowledge about data and therefore makes it compatible for other types of omics data outside of the genetics field.

**Figure 1.** Comparison of data size between bgen and HDF5 format. PLINK binary format .bed is shown as a reference.

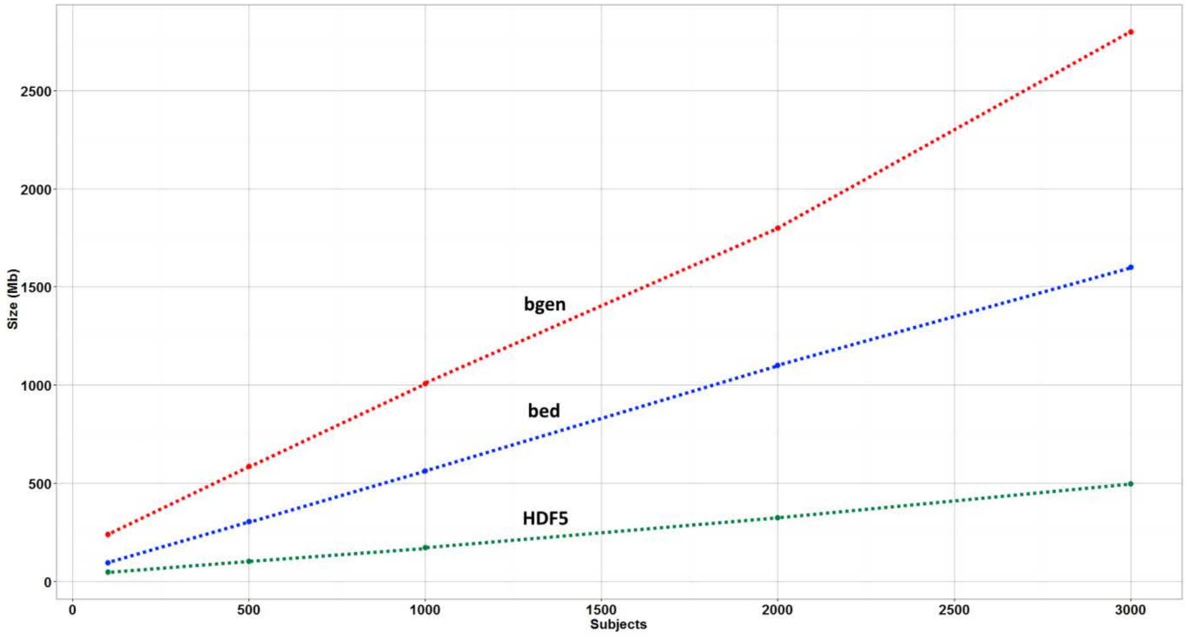

## References


1 Albert Hofman et al. “The Rotterdam Study: 2012 objectives and design update." In: European journal of epidemiology 26.8 (Aug. 2011), pp. 657

2 M. Arfan Ikram et al. “The Rotterdam Scan Study: design and update up to 2012." In: European journal of epidemiology 26.10 (Oct. 2011), pp. 811

3 C. D. Good et al. “A voxel-based morphometric study of ageing in 465 normal adult human brains." In: NeuroImage 14.1 Pt 1 (July 2001), pp. 21

4 Henri a Vrooman et al. “Multi-spectral brain tissue segmentation using automatically trained k-Nearest-Neighbor classification." In: NeuroImage 37.1 (Aug. 2007)

5 Stephen M. Smith et al. “Advances in functional and structural MR image analysis and implementation as FSL." In: NeuroImage 23 Suppl 1 (Jan. 2004)

6 https://www.hdfgroup.org/HDF5/doc/H5.format.html
